# Analysis of the Dual Use of Electronic Cigarettes and Conventional Tobacco According to the Survey on Alcohol and Other Drugs in the General Population in Spain (EDADES 2022)

**DOI:** 10.3390/ijerph22101507

**Published:** 2025-09-30

**Authors:** Javier Rubio-Serrano, Ileana Gefaell-Larrondo, Encarnación Serrano-Serrano, Eduardo Olano-Espinosa, César Minué-Lorenzo

**Affiliations:** 1 Foundation for Primary Care Biomedical Research and Innovation, Research Network on Chronicity, Primary Care and Health Promotion, 28003 Madrid, Spain; javier.rubio.externo@salud.madrid.org; 2 Federica Montseny Health Care Centre, Research Unit, Primary Care Managment, 28035 Madrid, Spain; 3 Los Fresnos Healthcare Center, 28850 Madrid, Spain; encarnacion.serrano@salud.madrid.org; 4 Concejalía de Salud Pública, Consumo y Bienestar Animal, Ayundatemiento de Alcorcón, 28921 Madrid, Spain; e.oeoeoeoe@gmail.com; 5 Perales del Río Health Care Centre, 28909 Madrid, Spain; cesaraugusto.minue@salud.madrid.org

**Keywords:** electronic cigarettes, tobacco, dual use, public health

## Abstract

Introduction: Electronic nicotine delivery systems present emerging challenges for public health. This study describes the use of electronic cigarettes (ECs) and dual consumption patterns in the Spanish population. Material and Methods: A descriptive cross-sectional study was conducted using data from the 2022 Survey on Alcohol and Other Drugs in the General Population in Spain (EDADES), targeting individuals aged 15–64. Variables included sociodemographic characteristics, dual consumption (defined as use of both EC and conventional tobacco, daily and/or within the last 30 days), perceived health status, and self-perceived risk of ECs or tobacco consumption. Analysis included descriptive statistics and multinomial logistic regression. Results: The study included 26,337 participants, (51% men). The prevalence of dual users in the population was 1.55% (95%CI: 1.40–1.70). Each additional year of age was associated with increased odds of exclusive tobacco use (aOR: 1.04; IC95%: 1.02–1.05). Compared to dual users, individuals with monthly incomes of 1000–1499 and 1500–2499 had higher odds of exclusive tobacco use (aOR 1.56; 95%CI: 1.03–2.34 and 1.90; 95%CI: 1.28–2.82, respectively). Dual use was associated with a ‘fair’ perceived health compared to exclusive EC users (aOR: 0.28; 95%CI: 0.13–0.60) and exclusive tobacco users (aOR: 0.62; 95%CI: 0.47–0.83), and with ‘poor/very poor’ health compared to exclusive tobacco users (OR: 0.43; 95%CI: 0.24–0.79). Among former EC users, 71% reported exclusive conventional tobacco use in the past 30 days. Conclusion: The prevalence of dual use in Spain was 1.55%. Dual users were younger than conventional tobacco smokers and, had lower income levels and poorer self-perceived health status compared to exclusive EC users.

## 1. Introduction

According to the World Health Organization (WHO) reports, tobacco consumption in Europe decreased by 9.3% between 2000 and 2020 [1]. In Spain, the National Health Surveys (NHS) also pointed towards an overall decrease in tobacco consumption, from 38.4% in 1987 to 18.3% in 2023 [2]. Several factors have contributed to this decline, such as the measures set out in the Framework Convention on Tobacco Control (FCTC) [3] regarding sales regulations, smoke-free environments, bans on advertising and sponsorship, and restrictions on the availability and accessibility of tobacco—especially for young people. These measures influenced the development of Spanish legislation in 2005 and 2010 [4,5]. They regulated tobacco consumption in workplaces and leisure venues, and together with other measures aimed at denormalising smoking and increasing fiscal pressure, reduced exposure to smoke, encouraged quit attempts, and were associated with declines in prevalence and acute events [6].

The prevalence of daily smoking among individuals over 15 years of age has declined to 16.6% [7], down from 19.8% in 2020 [8]. The 2024 EDADES survey, the results of which are available although we have not yet been able to obtain the microdata for analysis, conducted in the population aged 15 to 64 years, also shows a decrease in daily smoking, from 33.1% in 2022 to 25.8% in 2024 [9]. Consumption of ECs within the past 30 days rose from 1.5% in 2018 to 2.2% in 2022, reaching 4.6% in 2024 [9]. According to the biennial ESTUDES survey taken by secondary school students aged between 14 and 18 years, 17% had tried ECs at least once by 2014. By 2021, this percentage had risen to 44.3%, and in the most recent survey conducted in 2023, over half of the respondents (54.6%) reported having tried it. As many as 26.3% reported using ECs in the past 30 days [10]. The consumption of combustible cigarettes has declined over recent years, with a marked decrease since 2020. This trend has been accompanied by an increase in the use of electronic cigarettes, particularly among younger populations. In Europe, the Eurobarometer estimates that 6% of Europeans have used ECs, with 3% being regular users [11], and estimates that 82 million people were using ECs in 2021 [12].

The emergence of ECs entails significant challenges for tobacco control. The evidence regarding the risks and benefits of EC use is subject to debate and diverse interpretations. In clinical settings, when combined with counselling, ECs are more effective for smoking cessation than nicotine replacement therapy (NRT), although a considerable number of people are long-term users [13]. There is no evidence to suggest that it is more effective than varenicline [13]. Outside of clinical settings, its effectiveness in helping people quit conventional cigarettes is highly discussed and may even hinder the quitting process, according to several meta-analyses [14,15,16,17] that have also generated a wide controversy [18,19,20,21]. Other recent studies found that the use of e-cigarettes was associated with higher rates of conventional tobacco cessation [22,23,24]. However, as observed in clinical trials, a substantial proportion of individuals continue using e-cigarettes after quitting smoking [22].

Furthermore, it has been suggested that EC use could increase the likelihood of taking up smoking, potentially acting as a gateway. Some population-based studies have associated the use of e-cigarettes with a decline in conventional tobacco smoking [25]. A recent umbrella review showed an association between e-cigarette use and subsequent tobacco consumption among young people [26]. Another systematic review [27] suggests that “*At an individual level, people who vape appear to be more likely to go on to smoke than people who do not vape; however, it is unclear if these behaviours are causally linked. Very low certainty evidence suggests that youth vaping and smoking could be inversely related*”. However, the authors of this study also point out that *“Overall, the certainty of the evidence was judged to be very low. The evidence-base in this space has critical limitations and contradictions. The only thing that can be said with certainty is that the evidence is not certain”.* The dual user population is heterogeneous. Among the reasons that lead smokers to initiate e-cigarette use are the perception that these devices may help them quit smoking or that they are less harmful than conventional cigarettes. As for the reasons that explain continued use over time, commonly cited factors include the belief in reduced health risks, the possibility of reducing tobacco consumption, enjoyment, and flavour variety [28]. As their use to circumvent smoke-free space restrictions [29], use of new nicotine products fosters the adoption of various delivery methods depending on the environment and context. As a result, dual use of ECs and conventional tobacco may become the norm, especially in countries where EC use is more widely accepted [30]. In the UK, overall prevalence of dual use was 39%. Some studies report that between 19.6% and 59.4% of young smokers engage in dual use (ECs and conventional tobacco), which may decrease the motivation to quit, thereby sustaining nicotine addiction among smokers [14,15]. Dual users tend to have higher levels of dependence [31] and may face an increased risk of disease [32,33]. Although these meta-analyses have faced methodological criticism, the odds ratios for dual use remain higher than for exclusive tobacco use [34] (Lee, Farsalinos). Beyond methodological concerns, potential ties between some studies or authors and the tobacco or e-cigarette industry should also be considered when interpreting the evidence [35,36,37]

The latest WHO report [1] highlights the importance of monitoring EC use among adults and adolescents to better understand the factors driving it and how consumption patterns are evolving. Dual use of ECs and conventional cigarettes poses a public health challenge—not only due to the risks associated with smoking itself, but also because of the potential added harms of combined use and the continued dependence on nicotine. Therefore, this study aims to describe the use of ECs and dual use among the Spanish population aged 15–64 who participated in the 2022 Survey on Alcohol and Other Drugs in the General Population in Spain (EDADES) [38], as well as to analyze the associated sociodemographic characteristics and perceived health status.

## 2. Materials and Methods

### 2.1. Study Design and Population

A descriptive cross-sectional study was conducted using data from the 2022 EDADES survey, which targets the Spanish population aged 15 to 64 years.

The primary objective was to determine the prevalence of dual use of conventional tobacco and ECs in the Spanish population, specifically among EC users and tobacco smokers. As secondary objectives, we analyzed the associated factors to dual use and the transition patterns between dual use, exclusive EC or tobacco use, and cessation.

### 2.2. Variables

▪ Dual use was defined as individuals who use ECs and smoke tobacco, either daily and/or within the past 30 days.
▪ Conventional tobacco use was defined as smoking traditional tobacco products (either conventional cigarettes and/or hand-rolled tobacco) daily and/or within the past 30 days (Table S2).
▪ EC use was defined as daily consumption and/or use within the past 30 days.

Sociodemographic variables included:

▪ Sex (male/female).
▪ Age (in years and grouped in three: 15–25, 26–50, 51–65).
▪ Educational level (no education/primary, secondary, mid-level university students, upper-level university students).
▪ Employment status (working, no economic activity, retired, studying).
▪ Income level (≤EUR 999; EUR 1000–1499; EUR 1500–2499; EUR 2500–2999; ≥3000 or more).

Additional variables:

▪ Perceived health status: very good/good, average, and bad/very bad.
▪ Self-perceived risk of tobacco consumption or EC use: few or no problems, several or many problems.

### 2.3. Data Collection

The EDADES Survey (Alcohol and Other Drugs in the General Population in Spain) is coordinated by the Government Delegation for the National Drug Plan (DGPNSD) in collaboration with Spain’s regional governments. Since 1995, it has been conducted biennially, with 15 editions completed by 2024.

The survey collects data on the prevalence of alcohol, tobacco, sedative-hypnotics, opiates, and illicit psychoactive drug use, along with related topics such as user profiles, public perception of the risks associated with certain consumption behaviours, perceived awareness of the issue, and other relevant factors.

Data for this study was obtained through the Spanish Ministry of Health website upon request for research purposes. Variables related to tobacco and EC use were selected from the survey (Table S1).

### 2.4. Data Analysis

Sociodemographic characteristics and lifestyle factors were described using frequencies and percentages for qualitative variables and their median with interquartile range (IQR) for quantitative variables due to the non-normal distribution of the data.

A combined variable, dual use, was created to represent participants who reported using ECs and conventional tobacco within the past 30 days (Table S2).

The prevalence of dual consumption was calculated, followed by a bivariate analysis comparing dual users with those who exclusively smoke conventional tobacco and those who exclusively use ECs. Stratified analysis was performed by age group. The Kruskal–Wallis test was applied for non-parametric continuous variables, and Pearson’s chi-squared test for categorical variables.

A multinomial logistic regression model was conducted for explanatory variables that showed a significant association. The interactions between variables were tested to assess whether the effect of one variable on the dependent variable (in this case, the type of consumption) varies depending on the value of another variable.

All analyses considered a *p*-value < 0.05, the threshold for statistical significance. Analyses were conducted using R-Studio (version 4.4.2) [39].

## 3. Results

The 2022 EDADES survey included 26,337 participants aged 15–64; 38% (n = 10,003) reported daily use of conventional tobacco and/or ECs in the past 30 days. Among this group, 57.1% were male, and the median age was 37 [IQR: 26.0–48.0] years. A total of 74.3% had completed secondary education, 62.1% were employed, 30.5% reported a monthly income between EUR 1500 and EUR 2499, and 83.5% rated their health status as very good or good. Among all smokers and EC users, 94% (n = 9411) only smoked conventional tobacco, 4.09% (n = 409) were dual users, and 2% (n = 183) used ECs exclusively.

### 3.1. Prevalence and Characteristics of Dual Consumers

The prevalence of dual users in the population was 1.55% (95%CI: 1.40–1.70). Among all users of tobacco and/or ECs (n = 10,003), 4.09% (n = 409) were dual users. Dual users had a median age of 29 [IQR: 22.0–40.0] years, and 53% were male. A total of 75.1% had completed secondary education, 52.8% were employed, and 21.8% reported an income between EUR 1500 and EUR 2499 per month. Among all EC users, 69.08% (95% CI: 66.1–73.5%) were dual users of EC and combustible tobacco, 18.41% were former conventional tobacco smokers, and 7.43% had never smoked tobacco.

Compared to exclusive conventional tobacco or EC users, dual users were more likely to be economically inactive (23.0% vs. 20.9% vs. 14.8%) and have an income below EUR 999 (11.0% vs. 7.1% vs. 3.8%).

Among dual users, 15.9% reported a fair perceived health status, compared to 4.4% of exclusive EC users and 14.1% of exclusive conventional tobacco users, and a poor or very poor perceived health in 3.2% vs. 0% vs. 2.0% of cases.

Participants expressed a higher perceived risk in smoking tobacco, which increased to 93.4% among exclusive e-cigarette users. Dual users perceived both tobacco (17.6%) and e-cigarettes (49.9%) as lower risk compared to other consumers (Table 1).

Patterns of use showed that 46.9% (n = 192) of dual users smoked tobacco daily and had used ECs in the last 30 days, but not daily. Daily use of both ECs and tobacco was reported by 36.4% (n = 149), while 8.6% (n = 35) had used both in the last 30 days, but without daily consumption of either. A total of 4.9% (n = 20) used ECs daily and had smoked tobacco in the last 30 days (Table 2).

### 3.2. Impact of Factors Associated with EC Use and Dual Use (Figure 1, Table S4)

Being a dual user was associated with a ‘fair’ perceived health status compared to exclusive e-cigarette users, aOR of 0.28 (95% CI: 0.13–0.60).

By comparing exclusive tobacco users to dual users, we find that with each additional year of age, the likelihood of using only tobacco increased by 4%, aOR 1.04 (95%CI: 1.02–1.05), compared to dual consumption. Having an income between EUR 1000–1499 and EUR 1500–2499 was associated with exclusive tobacco use compared to dual use: aOR 1.56; 95%CI: 1.03–2.34) and 1.9; 95%CI: 1.28–2.82), respectively. Dual users reported a regular perceived health status (aOR 0.62; 95%CI: 0.47–0.83) and bad/very bad (aOR 0.43; 95%CI: 0.24–0.79) with respect to exclusive tobacco consumption.

Similarly, with each additional year of age, the likelihood of being an exclusive tobacco user rather than an exclusive EC user increased by 4% (aOR 1.04; 95%CI: 1.02–1.06). Being a student was associated with exclusive EC use compared to only consuming tobacco (aOR 0.64; 95%CI: 0.42–0.98). A ‘fair’ perceived health status was associated with exclusive tobacco when compared to a ‘very good/good’ perceived health status (aOR 2.26; 95%CI: 1.09–4.67).

**Figure 1:**
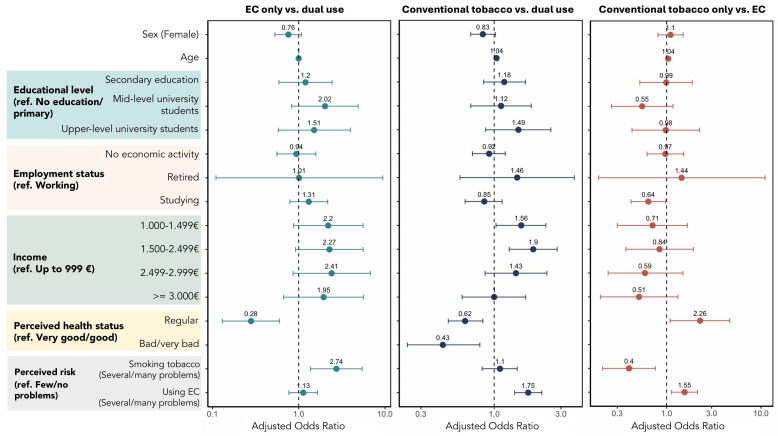
Factors associated with dual smoking compared to EC-only and tobacco-only use: (**left**) EC usage versus dual smoking; (**middle**) conventional tobacco smoking versus dual usage; (**right**) conventional tobacco smoking versus EC usage.

The perceived risk of smoking a pack of cigarettes a day as severe or causing many problems was associated with exclusive EC use compared to dual use (aOR 2.74; 95%CI: 1.37–5.45) and exclusive tobacco use (aOR 0.40; 95%CI: 0.21–0.76). A perceived risk of EC use as severe or causing many problems was associated with exclusive tobacco use compared to dual use (aOR 1.75; 95%CI: 1.40–2.19) and exclusive EC use (aOR 1.55; 95%CI: 1.13–2.13) (Figure 1).

### 3.3. Changes in E-Cigarette Smokers

Among the total number of EC users (n = 592) in the surveys, 18.41% were former conventional tobacco smokers, and 7.43% had never tried conventional tobacco. Similarly, the survey included 2275 former EC users, 22% of whom had completely quit both ECs and tobacco. Among former EC users, 1617 (71.08%) now exclusively smoked conventional tobacco. Among the total number of tobacco smokers (n = 9820), 16.46% were former EC users, whereas 4.16% (409) were dual users.

## 4. Discussion

Main findings: According to the National EDADES survey, the prevalence of dual use in the Spanish population is 1.55%. A total of 69.08% (95% CI: 66.1–73.5%) of EC users were also conventional tobacco smokers, and 4.3% of tobacco smokers are dual consumers. Dual users were younger than conventional tobacco smokers, were more likely to be economically inactive, and had lower monthly incomes compared to both conventional tobacco and EC users. A perceived risk of EC use as severe or causing many problems was associated with exclusive tobacco use compared to dual use and exclusive EC use.

### 4.1. Comparison with Pre-Existing Literature

The prevalence of dual use of tobacco and ECs varies widely across different studies and populations. According to a systematic review, the prevalence of dual use among young people is 4% [40]. In Spain, in a 2014 study using data from the Omnibus survey, 10.3% (IC95%: 8.6–12.4) of the Spanish adult population reported having tried ECs at least once. Among EC users, 57.2% also smoked combustible tobacco, while 14.8% were former conventional cigarette smokers [41]. The prevalence of dual use of tobacco and ECs varies widely across different studies and populations. A survey conducted across 12 European countries revealed substantial differences in e-cigarette and dual-use prevalence between countries. The proportion of dual users in Europe exceeded 50%, ranging from 23% in Germany to 87% in Spain and 90% in Romania [29]. In some contexts, dual use appeared to serve as a strategy to circumvent smoke-free legislation, while in others, e-cigarettes were used as a more affordable alternative in countries where conventional tobacco is more expensive. In Poland, 15.2% of respondents had used e-cigarettes in the past 30 days, and 5.9% reported daily use. Among nicotine product users, 10% engaged in dual use of conventional and electronic cigarettes, with an additional 9% using both conventional cigarettes and heated tobacco products [42]. In Sweden, 2% of respondents reported using e-cigarettes, two-thirds of whom (66.7%] were dual users—a proportion closely aligned with findings from the EDADES survey [43]. In Ireland, dual use has reached 3.1% [44]. In the United Kingdom, public health authorities have actively promoted e-cigarettes as a smoking cessation tool. In England, dual smoking among conventional smokers accounts for 39%, and the percentage of dual smoking among EC users is around 70% [30].

Outside Europe, the PATH survey in the US showed that 20.8% of tobacco smokers also use ECs [45]. Nagel reports a dual-use prevalence of 2.3% overall; 3.9% among youth, with 3.6% in men and 1.1% in women. In our study, we found the figure to be 4.16%. In China, the prevalence of EC use was 1.6%, of which more than 90% were dual users [46]. In Korea, up to 85% of e-cigarette users also smoked tobacco [47].

In our analysis, there were no differences between sexes in dual smokers. According to the PATH survey, young people, women, non-Latino whites, and those with higher educational levels were at greater risk for dual consumption [45].

Another multi-wave analysis of the PATH survey examines sex differences in dual users. These were more often men. Women were more dependent on cigarettes, whereas men were more frequent users of ECs [48]. Other studies also found that men engage in dual consumption more frequently [43,49,50].

Dual users have greater nicotine dependence, higher alcohol and cannabis use, and are more likely to have used non-medical opioids [51].

In our study, as age increases, the likelihood of exclusively using combustible tobacco also increases (details of stratified analysis are available in Table S3). Other studies confirm a lower dual consumption with age, although dual smokers are not usually the youngest but rather in their twenties or early thirties [30,45,49,50].

While some studies, such as Assari’s, find no link between dual consumption and economic status, they do observe a correlation with higher education levels; others, such as Hedman’s, report different findings. In China and Korea, EC consumption is associated with higher economic levels [46,47], unlike in the United Kingdom [30,50].

In our study, only 1.43% of ex-smokers reported using ECs. The prevalence of vaping among individuals in the UK who quit conventional tobacco smoking over one year ago has risen from 1.9% in 2013 to 20.4% in 2024 [52].

Interestingly, 71% of individuals who have had some contact with e-cigarettes in the past—but who no longer use them, either because they quit or were never regular users—now smoke conventional tobacco. However, the EDADES survey does not allow us to hypothesize the reasons underpinning this shift. For instance, the survey does not define a lower end limit for smoking or vaping, like having smoked 100 cigarettes or a minimal frequency of continuous usage of ECs in a given time lapse. Whereas smokers typically begin with an experimental phase that often leads to regular use, users and former users of electronic cigarettes display a much wider range of behaviours: some experiment without continuing, others maintain regular use, some transition to conventional cigarettes, others use them as a tool to quit smoking, and some try them out of curiosity, the ability to use them in smoke-free spaces, or due to the perception of lower health risks. Based on an analysis of the PATH survey in the U.S., Kaplan [16] estimates that for every beneficial transition—such as switching from conventional cigarettes to exclusive EC use or quitting smoking while using ECs as a support tool—there were 2.15 harmful transitions (starting to use ECs or switching from ECs to exclusively smoking combustible cigarettes).

Dual smokers reported poorer health compared to those who smoked only tobacco or ECs. Dual use has been associated with a higher risk of respiratory conditions such as chronic obstructive pulmonary disease and asthma, as well as cardiovascular and metabolic diseases, along with poorer overall health outcomes [32,33]. In Spain, an analysis of the ESTUDES survey [53] found that individuals who had ever used ECs were nearly twice as likely to smoke conventional cigarettes further down the line. Dual smokers tend to consume more cigarettes daily, use nicotine-containing ECs more frequently, and start smoking at a younger age. Several meta-analyses and systematic reviews indicate that EC use is linked to an increased likelihood of progressing to regular cigarette smoking [27,54,55,56].

After the WHO Framework Convention on Tobacco Control, the Spanish government introduced Law 28/2005 on health measures against smoking. Five years later, it was reformed and became one of the most advanced tobacco control laws in Europe. The law enabled protection of non-smokers from second-hand smoke in public places and workplaces, extended the ban on direct advertising, promotion, and sponsorship, and regulated points of sale. It also contributed to progress in the implementation of the MPOWER strategy.

However, over the past 15 years, there has been little further progress in tobacco control policy. Outstanding challenges include the need to strengthen cessation support services, improve enforcement of the law in semi-enclosed public spaces, extend smoke-free areas such as outdoor terraces and other public spaces, adopt plain packaging (already implemented in many countries), and significantly and progressively increase tobacco taxes to levels comparable to other countries. This includes aligning the taxation of roll-your-own tobacco and heated tobacco products with that of conventional cigarettes.

Some of these measures, along with bringing e-cigarettes and related products under the same regulatory framework as traditional tobacco, are part of Spain’s Comprehensive Plan for Tobacco Prevention and Control, which is intended to support the development of a new legislative framework [57].

### 4.2. Strengths and Limitations:

The EDADES surveys are designed to provide a highly representative sample of the Spanish population. This is supported by its large sample size and use of random sampling, making the study more accurate while minimizing selection bias.

However, it is important to consider the limitations inherent to cross-sectional surveys. Although the surveys are conducted by trained researchers, responses may be affected by information bias, particularly due to the subjective nature of certain questions—such as income level, ex-smoker status, and self-perceived health status. Similarly, we cannot draw causal conclusions about changes in smoking habits among former EC users, as their motivations and decision-making factors were not documented.

Although the most recent EDADES survey was held in 2024, and preliminary results have been published by the Ministry of Health, the necessary microdata to perform a rigorous quantitative analysis—comparable to that carried out with the 2022 data—has not yet been published. Future studies will be able to make trend analyses of dual use between the different surveys [9].

### 4.3. Implications

To our knowledge, this is the first study of a Spanish population to estimate the prevalence of dual smoking, defined as the use of both ECs and conventional tobacco within the past 30 days. Our findings suggest that, despite ECs being promoted as a way to quit conventional tobacco [13], most EC users are dual users. Therefore, legislation and future prevention and health promotion policies should take into account the behaviours of smokers and nicotine users at a population level.

### 4.4. Future Research

Further studies are needed to look into the factors and motivations behind switching from ECs to conventional tobacco, dual use, and the proportion of smokers who quit nicotine entirely using ECs. These motivations could be addressed through survey questions or by conducting targeted questionnaires and qualitative research among populations at the heart of these shifts in nicotine consumption. Similarly, it is of utmost importance to tackle the increasing use of ECs and dual use—particularly among specific age groups—by conducting studies that assess potential interventions to curb these behaviours.

## 5. Conclusions

In 2022, dual users of ECs and tobacco accounted for 4% of smokers in the Spanish population. Dual users tend to be younger than conventional tobacco smokers, and, compared to exclusive EC users, they have lower income levels and poorer self-perceived health status. A perceived risk of EC use as severe or causing many problems was associated with exclusive tobacco use compared to dual use and exclusive EC use.

Although traditional tobacco use is waning, it is of utmost importance to address the rising prevalence of ECs and dual use—particularly among specific age groups—through studies that assess potential interventions.

## Figures and Tables

**Table 1:** Sociodemographic characteristics and lifestyle by type of consumption.

Variables	Dual Consumer (n = 409)	EC Only (n = 183)	Tobacco Only (n = 9411)	Overall (n = 10,003)	*p*
Sex (male)	217 (53.1)	109 (59.6)	5384 (57.2)	5710 (57.1)	0.199 ^a^
Age	29.0 [22.0–40.0]	26.0 [21.0–37.0]	37.0 [27.0–48.0]	37.0 [26.0–48.0]	<0.001 ^b^
Age group					
15–25 years	166 (40.6)	83 (45.4)	2022 (21.5)	2271 (22.7)	<0.001 ^a^
26–40 years	141 (34.5)	63 (34.4)	3643 (38.7)	3847 (38.5)	
41–65 years	102 (24.9)	37 (20.2)	3746 (39.8)	3885 (38.8)	
Educational level					
No education/primary	47 (11.5)	13 (7.1)	910 (9.7)	970 (9.7)	0.067 ^a^
Secondary	307 (75.1)	132 (72.1)	6991 (74.3)	7430 (74.3)	
Mid-level university students	31 (7.5)	23 (12.6)	693 (7.4)	747 (7.5)	
Upper-level university students	24 (5.9)	14 (7.7)	799 (8.5)	837 (8.4)	
Employment status					
Working	216 (52.8)	99 (54.1)	5898 (62.7)	6213 (62.1)	<0.001 ^a^
No economic activity	94 (23.0)	27 (14.8)	1966 (20.9)	2087 (20.9)	
Retired	5 (1.2)	1 (0.5)	347 (3.7)	353 (3.5)	
Studying	89 (21.8)	53 (29.0)	1098 (11.7)	1240 (12.4)	
Income					
Up to EUR 999	45 (11.0)	7 (3.8)	672 (7.1)	724 (7.2)	<0.001 ^a^
From EUR 1000 to 1499	63 (15.4)	28 (15.3)	1595 (16.9)	1686 (16.9)	
From EUR 1500 to 2499	89 (21.8)	46 (25.1)	2913 (31.0)	3048 (30.5)	
From EUR 2500 to 2999	29 (7.1)	17 (9.3)	714 (7.6)	760 (7.6)	
3000 or more	27 (6.6)	14 (7.7)	493 (5.2)	534 (5.3)	
Perceived health status					
Very good/good	326 (79.7)	175 (95.6)	7850 (83.4)	8351 (83.5)	<0.001 ^c^
Regular	65 (15.9)	8 (4.4)	1324 (14.1)	1397 (14.0)	
Bad/very bad	13 (3.2)	0 (0)	192 (2.0)	205 (2.0)	
Perceived risk of smoking one pack of cigarettes per day					
Few or no problems	72 (17.6)	11 (6.0)	1133 (12.0)	1216 (12.2)	<0.001 ^c^
Several or many problems	333 (81.4)	171 (93.4)	8150 (86.6)	8654 (86.5)	
Perceived risk of using EC					
Few or no problems	204 (49.9)	79 (43.2)	3021 (32.1)	3304 (33.0)	<0.001 ^c^
Several or many problems	175 (42.8)	95 (51.9)	5027 (53.4)	5297 (53.0)	

^a^: *t*-test; ^b^: Wilcoxon-ram-sum test; ^c^: chi-squared test.

**Table 2:** Patterns of use of tobacco or e-cigarettes in dual users.

Patterns of Use in Dual Users	n	%
Smoked tobacco daily, used ECs in the last 30 days	192	46.9
Daily users of both ECs and tobacco	149	36.4
Used both ECs and tobacco in the last 30 days	35	8.6
Daily EC use, smoked tobacco in the last 30 days	20	4.9
Dual users, but frequency of use could not be determined	13	3.2
Total	409	100%

## Data Availability

The data is freely available under request, through the Ministry of Health platform. https://pnsd.sanidad.gob.es/profesionales/sistemasInformacion/sistemaInformacion/encuestas_EDADES.htm (accessed on 22 January 2025).

## Supplementary Materials

The following supporting information can be downloaded at: https://www.mdpi.com/article/10.3390/ijerph22101507/s1, Table S1: Selected variables from the EDADES survey; Table S2: Definition of smoker, EC user, and dual user. Table S3: Sociodemographic characteristics and lifestyle by type of consumption stratified by age group. Table S4: Impact of sociodemographic factors and lifestyle based on type of consumption, EC use versus dual use, conventional tobacco use versus dual use, and conventional tobacco use versus EC use.

## Abbreviations

The following abbreviations are used in this manuscript:

EC	Electronic cigarette
WHO	World Health Organization
NHS	National Health Surveys
FCTC	Framework Convention on Tobacco Control
EDADES	Survey on Alcohol and Other Drugs in the General Population in Spain
DGPNSD	Government Delegation for the National Drug Plan
