# Supplementary material for: Analysis of the Dual Use of Electronic Cigarettes and Conventional Tobacco According to the Survey on Alcohol and Other Drugs in the General Population in Spain (EDADES 2022)
Source: Int J Environ Res Public Health. 2025 Sep 30;22(10):1507. doi: 10.3390/ijerph22101507 (PMC12564643; doi:10.3390/ijerph22101507)
Supplement: Supplementary file 1 [file ijerph-22-01507-s001.zip › Table S1. Selected variables from the EDADES survey.pdf]

Question number	Question text	Possible answers
D1	Sex	- Male<br>- Female
D2	How old are you?	Quantitative variable
D5	What is the highest level of education you have completed or passed? Classify people according to the highest level or course passed. If you're unsure where to place certain levels of education, classify according to the official qualifications needed to enrol on these courses—for example, assign a 3 if they have completed Compulsory Secondary Education or if a Secondary School Completion Certificate is required, or a 4 if completion of a Bachelor's Degree is required.	- No formal education or not having completed primary education<br>- Completed primary education<br>- Secondary education 1st stage<br>- Secondary education 2nd stage<br>- University education<br>- Higher level university education<br>- DK/NA
D9	Next I'm going to ask you some questions about your working life. Which of the following situations were you in LAST WEEK? We are referring to the last full week, from last Sunday to the previous Monday.	- Working<br>- Employed, but temporarily on a leave of absence<br>- Unemployed person who has previously worked<br>- Unemployed person looking for his/her first job<br>- Retired<br>- Total permanent disability<br>- Studying<br>- Homemaker<br>- Another situation with no economic activity<br>- DK/NA
D16	I'm going to ask you what your approximate net monthly household income currently is.	- Up to €499<br>- From €500 to €999<br>- From €1,000 to €1,499<br>- From €1500 to €1999<br>- From €2,000 to €2,499<br>- From €2,500 to €2,999<br>- From €3,000 to €4,999<br>- €5,000 or more<br>- DK/NA
S1	Generally, you would say that your health status is...	- Very Good/good<br>- Average<br>- Bad/very bad<br>- DK/NA
T1_1	Please indicate whether you have ever smoked a cigarette or other type of tobacco, even just one or two puffs.	- Yes<br>- No
T1_2	Please indicate whether you have smoked a cigarette or other type of tobacco, even if it was only one or two puffs, within the last 12 months	- Yes<br>- No
T1_3	Please indicate whether you have smoked a cigarette or other type of tobacco, even if it was only one or two puffs,	- Yes<br>- No

	within the last 30 days	
T1_4	Please indicate whether you have smoked a cigarette or other type of tobacco, even if it was only one or two puffs, daily within the last 30 days	- Yes<br>- No
EC1_1	Please indicate whether you have ever smoked e-cigarettes (vaping).	- Yes<br>- No
EC1_2	Please indicate whether you have smoked e-cigarettes (vaping), within the last 12 months.	- Yes<br>- No
EC1_3	Please indicate whether you have smoked e-cigarettes (vaping), within the last 30 days.	- Yes<br>- No
EC1_4	Please indicate whether you have smoked e-cigarettes (vaping), daily within the last 30 days.	- Yes<br>- No
R1_1	Perceived risk of smoking one pack of cigarettes per day	- Few or no problems<br>- Several or many problems
R1_2	Perceived risk of using e-cigarettes	- Few or no problems<br>- Several or many problems
