# Supplementary material for: Analysis of the Dual Use of Electronic Cigarettes and Conventional Tobacco According to the Survey on Alcohol and Other Drugs in the General Population in Spain (EDADES 2022)
Source: Int J Environ Res Public Health. 2025 Sep 30;22(10):1507. doi: 10.3390/ijerph22101507 (PMC12564643; doi:10.3390/ijerph22101507)
Supplement: Supplementary file 1 [file ijerph-22-01507-s001.zip › Table S2. Definition of smokers, users of EC and dual users.pdf]

Table S2. Definition of smokers, users of ECs, and dual users.

Survey variables related to EC usage:

- CE1_1: Have you ever used ECs in your life?
- CE1_2: Have you used ECs in the last 12 months?
- CE1_3: Have you used ECs in the last 30 days?
- CE1_4: Have you used ECs daily in the last 30 days?

CE1_1	CE1_2	CE1_3	CE1_4	New variable	Variables used in the study
No	No	No	No	Non-user of ECs	Non-user of ECs
Yes	No	No	No	Ex-user of ECs	Former EC user
Yes	Yes	No	No	EC user in the last 12 months	Not applicable
Yes	Yes	Yes	No	EC user in the last 30 days	EC User
Yes	Yes	Yes	Yes	Daily EC user	

Survey variables related to tobacco consumption:

- T1_1 Have you ever used cigarettes or other types of tobacco in your life?
- T1_2 Have you used cigarettes or other types of tobacco in the last 12 months?
- T1_3 Have you used cigarettes or other types of tobacco in the last 30 days?
- T1_4 Have you ever consumed cigarettes or other types of tobacco daily in the last 30 days?

T1_1	T1_2	T1_3	T1_4	New variable	Variables used in the study
No	No	No	No	Non-smoker of tobacco	Non-user of tobacco
Yes	No	No	No	Ex-smoker of tobacco	Former tobacco user
Yes	Yes	No	No	Smoker in the last 12 months of tobacco	Not applicable
Yes	Yes	Yes	No	Smoker in the last 30 days of tobacco	Tobacco smoker
Yes	Yes	Yes	Yes	Daily smoker of tobacco	

Dual users = EC User + Tobacco smoker

EC: electronic cigarette; not applicable refers to a variable that we finally did not include in the study.
