# Supplementary material for: Analysis of the Dual Use of Electronic Cigarettes and Conventional Tobacco According to the Survey on Alcohol and Other Drugs in the General Population in Spain (EDADES 2022)
Source: Int J Environ Res Public Health. 2025 Sep 30;22(10):1507. doi: 10.3390/ijerph22101507 (PMC12564643; doi:10.3390/ijerph22101507)
Supplement: Supplementary file 1 [file ijerph-22-01507-s001.zip › Table S3. Sociodemographic characteristics and lifestyle by type of consumption stratified by age group.pdf]

Table S3. Sociodemographic characteristics and lifestyle by type of consumption stratified by age group.

	15-25 years				26-40 years				41-65 years			
	**Dual consumer (n=166)**	**ECs only (n=83)**	**Tobacco only (n=2022)**	***p***	**Dual consumer (n=141)**	**ECs only (n=63)**	**Tobacco only (n=3643)**	***p***	**Dual consumer (n=102)**	**ECs only (n=37)**	**Tobacco only (n=3746)**	***p***
**Sex (male)[1]**	86 (51.8)	52 (62.7)	1,123 (55.5)	0.268[a]	79 (56.0)	33 (52.4)	2,105 (57.8)	0.640[a]	52 (51.0)	24 (64.9)	2,156 (57.6)	0.273[a]
**Educational level[1]**												
No education/primary	21 (12.7)	9 (10.8)	160 (7.9)	0.321[a]	14 (9.9)	3 (4.8)	265 (7.3)	0.032[a]	12 (11.8)	1 (2.7)	485 (12.9)	0.019[a]
Secondary	137 (82.5)	67 (80.7)	1,665 (82.3)		99 (70.2)	43 (68.3)	2,633 (72.3)		71 (69.6)	22 (59.5)	2,693 (71.9)	
Mid-level university students	5 (3.0)	4 (4.8)	102 (5.0)		16 (11.3)	12 (19.0)	338 (9.3)		10 (9.8)	7 (18.9)	253 (6.8)	
Upper-level university students	3 (1.8)	3 (3.6)	91 (4.5)		12 (8.5)	4 (6.3)	400 (11.0)		9 (8.8)	7 (18.9)	308 (8.2)	
**Employment status[1]**												
Working	51 (30.7)	22 (26.5)	732 (36.2)	0.216[a]	98 (69.5)	46 (73.0)	2,802 (76.9)	0.038[a]	67 (65.7)	31 (83.8)	2,364 (63.1)	0.180[a]
No economic activity	27 (16.3)	11 (13.3)	307 (15.2)		39 (27.7)	11 (17.5)	682 (18.7)		28 (27.5)	5 (13.5)	977 (26.1)	
Retired	0 (0)	0 (0)	0 (0)		0 (0)	0 (0)	0 (0)		5 (4.9)	1 (2.7)	347 (9.3)	
Studying	84 (50.6)	48 (57.8)	954 (47.2)		4 (2.8)	5 (7.9)	135 (3.7)		1 (1.0)	0 (0)	9 (0.2)	
**Income[1]**												
Up to EUR 999	14 (8.4)	4 (4.8)	131 (6.5)	0.337[a]	13 (9.2)	1 (1.6)	228 (6.3)	0.003[a]	18 (17.6)	2 (5.4)	313 (8.4)	0.009[a]
From EUR 1,000 to 1,499	15 (9.0)	7 (8.4)	258 (12.8)		34 (24.1)	14 (22.2)	646 (17.7)		14 (13.7)	7 (18.9)	691 (18.4)	
From EUR 1,500 to 2,499	36 (21.7)	22 (26.5)	460 (22.7)		26 (18.4)	16 (25.4)	1,267 (34.8)		27 (26.5)	8 (21.6)	1,186 (31.7)	
From EUR 2,500 to 2,999	5 (3.0)	6 (7.2)	126 (6.2)		17 (12.1)	6 (9.5)	310 (8.5)		7 (6.9)	5 (13.5)	278 (7.4)	
3,000 or more	5 (3.0)	6 (7.2)	94 (4.6)		12 (8.5)	3 (4.8)	197 (5.4)		10 (9.8)	5 (13.5)	202 (5.4)	
**Perceived health status[1]**												
Very good/good	142 (85.5)	82 (98.8)	1,886 (93.3)	0.004[a]	111 (78.7)	59 (93.7)	3,265 (89.6)	<0.001[a]	73 (71.6)	34 (91.9)	2,699 (72.1)	0.196[a]
Regular	19 (11.4)	1 (1.2)	111 (5.5)		23 (16.3)	4 (6.3)	320 (8.8)		23 (22.5)	3 (8.1)	893 (23.8)	
Bad/very bad	2 (1.2)	0 (0)	13 (0.6)		6 (4.3)	0 (0)	40 (1.1)		5 (4.9)	0 (0)	139 (3.7)	
**Perceived risk of smoking one pack of cigarettes per day[1]**												
Few or no problems	32 (19.3)	7 (8.4)	233 (11.5)	0.028[a]	24 (17.0)	3 (4.8)	491 (13.5)	0.135[a]	16 (15.7)	1 (2.7)	409 (10.9)	0.252[a]
Several or many problems	132 (79.5)	76 (91.6)	1,764 (87.2)		116 (82.3)	60 (95.2)	3,103 (85.2)		85 (83.3)	35 (94.6)	3,283 (87.6)	
**Perceived risk of smoking ECs[1]**												
Few or no problems	79 (47.6)	37 (44.6)	738 (36.5)	0.027[a]	71 (50.4)	26 (41.3)	1,213 (33.3)	<0.001[a]	54 (52.9)	16 (43.2)	1,070 (28.6)	<0.001[a]
Several or many problems	74 (44.6)	41 (49.4)	1,070 (52.9)		63 (44.7)	35 (55.6)	1,958 (53.7)		38 (37.3)	19 (51.4)	1,999 (53.4)	

EC: electronic cigarette; 1: n (%); a: Chi–squared test.
