# Supplementary material for: Analysis of the Dual Use of Electronic Cigarettes and Conventional Tobacco According to the Survey on Alcohol and Other Drugs in the General Population in Spain (EDADES 2022)
Source: Int J Environ Res Public Health. 2025 Sep 30;22(10):1507. doi: 10.3390/ijerph22101507 (PMC12564643; doi:10.3390/ijerph22101507)
Supplement: Supplementary file 1 [file ijerph-22-01507-s001.zip › Table S4. Impact of sociodemographic factors and lifestyle based on type of consumption_ EC use versus dual use, conventional tobacco use versus dual use, and conventional .pdf]

Table S4. Impact of sociodemographic factors and lifestyle based on type of consumption: EC use versus dual use, conventional tobacco use versus dual use, and conventional tobacco use versus EC use.

Variables	ECs only vs. dual use			Conventional tobacco only vs. dual use			Conventional tobacco only vs. ECs		
	aOR	CI95%	*p*	aOR	CI95%	*p*	aOR	CI95%	*p*
**Sex**									
Male (ref.)	1			1			1		
Female	0.76	0.53–1.08	0.126	0.83	0.68–1.02	0.079	1.10	0.81–1.50	0.525
**Age**	1.00	0.98–1.02	0.703	1.04	1.02–1.05	<0.001	1.04	1.02–1.06	<0.001
**Educational level**									
No education/primary (ref.)	1			1			1		
Secondary	1.20	0.59–2.44	0.623	1.18	0.84–1.67	0.338	0.99	0.52–1.87	0.975
Mid-level university students	2.02	0.83–4.90	0.119	1.12	0.68–1.84	0.659	0.55	0.26–1.17	0.123
Upper-level university students	1.51	0.58–3.98	0.400	1.49	0.87–2.54	0.143	0.98	0.43–2.24	0.968
**Employment status**									
Working (ref.)	1			1			1		
No economic activity	0.94	0.56–1.58	0.826	0.92	0.70–1.20	0.532	0.97	0.62–1.52	0.901
Retired	1.01	0.11–9.45	0.991	1.46	0.57–3.74	0.429	1.44	0.19–11.03	0.723
Studying	1.31	0.79–2.17	0.291	0.85	0.62–1.14	0.275	0.64	0.42–0.98	0.039
**Income**									
Up to EUR 999 (ref.)	1			1			1		
From EUR 1,000 to 1,499	2.20	0.87–5.58	0.098	1.56	1.03–2.34	0.034	0.71	0.30–1.66	0.428
From EUR 1,500 to 2,499	2.27	0.92–5.59	0.076	1.90	1.28–2.82	0.001	0.84	0.37–1.92	0.680
From EUR 2,500 to 2,999	2.41	0.86–6.77	0.096	1.43	0.86–2.38	0.165	0.59	0.24–1.49	0.267
3,000 or more	1.95	0.67–5.68	0.221	1.00	0.59–1.68	0.988	0.51	0.20–1.32	0.167
**Perceived health status**									
Very good/good (ref.)	1			1			1		
Regular	0.28	0.13–0.60	0.001	0.62	0.47–0.83	0.001	2.26	1.09–4.67	0.028
Bad/very bad	–	–	–	0.43	0.24–0.79	0.007	–	–	–
**Perceived risk of smoking one pack of cigarettes per day**									
Few or no problems (ref.)	1			1			1		
Several or many problems	2.74	1.37–5.45	0.004	1.10	0.82–1.46	0.537	0.40	0.21–0.76	0.005
**Perceived risk of using ECs**									
Few or no problems (ref.)	1			1			1		
Several or many problems	1.13	0.77–1.65	0.532	1.75	1.40–2.19	<0.001	1.55	1.13–2.13	0.006

aOR: adjusted Odds Ratio; EC: electronic cigarette; CI95%: 95% confidence interval.
